# Hyperbilirubinemia after surgical repair for acute type a aortic dissection: A propensity score-matched analysis

**DOI:** 10.3389/fphys.2022.1009007

**Published:** 2022-10-10

**Authors:** Zhigang Wang, Jingfang Xu, Xiaofeng Cheng, Lifang Zhang, Dongjin Wang, Jun Pan

**Affiliations:** ^1^ Department of Cardio-Thoracic Surgery, Affiliated Drum Tower Hospital, Medical School of Nanjing University, Nanjing, China; ^2^ Department of Nephrology, Nanjing Drum Tower Hospital Clinical College of Nanjing University of Chinese Medicine, Nanjing, China; ^3^ Department of Cardio-Thoracic Surgery, Nanjing Drum Tower Hospital Clinical College of Traditional Chinese and Western Medicine, Nanjing University of Chinese Medicine, Nanjing, China; ^4^ Department of Psychiatry, The First Affiliated Hospital, Zhengzhou University, Zhengzhou, China

**Keywords:** type A aortic dissection, hyperbilirubinemia, risk factors, cardiopulmonary bypass, outcome

## Abstract

**Background:** Inflammation and oxidative stress are known to participate in the pathogenesis of hyperbilirubinemia. It has been known that acute type A aortic dissection (ATAAD) surgical repair often associates with complications which might affect the long-term prognosis. However, the clinical significance of postoperative hyperbilirubinemia (PH) has not been evaluated. Here in this study, we examined the incidence, risk factors, and prognosis of PH after ATAAD surgery.

**Methods:** This retrospective study enrolled a total of 970 patients who received ATAAD surgical repair from January 2014 to December 2019. PH was defined as serum total bilirubin >3.0 mg/dl within the first week after the surgery. Propensity score matching was used to reduce selection bias and eliminate potential confounding factors. Kaplan–Meier survival and Cox proportional hazards regression analyses were conducted to explore the association between PH and postoperative long-term survival.

**Results:** Development of PH (183 patients) was associated with a higher 30-Day mortality (20.8% vs. 9.0%, *p* < 0.001). Advanced age [odds ratio (OR) 1.538, *p* = 0.006], higher baseline total bilirubin level (OR 1.735, *p* = 0.026), preoperative pericardial tamponade (OR 3.192, *p* = 0.024), prolonged cardiopulmonary bypass (CPB) duration (OR 2.008, *p* = 0.005), and elevated postoperative central venous pressure (CVP) level (OR 2.183, *p* < 0.001) were associated with PH. The Kaplan-Meier analysis showed patients who developed PH were associated with poor long-term survival (*p* = 0.044). Cox analysis showed that the presence of PH (hazard ratio 2.006, *p* = 0.003) was an independent risk factor for increased mortality.

**Conclusion:** PH is a common complication in patients undergoing ATAAD surgical repair that associates with worse short- and long-term prognosis. Our data indicated that age, preoperative total bilirubin level, pericardial tamponade, CPB duration, and postoperative CVP level were risk factors for the development of PH.

## Introduction

Acute type A acute aortic dissection (ATAAD) is a cardiovascular emergency event that often associated with lethal result if left untreated. Despite recent advances of endovascular interventions and improvement of postoperative management, open surgery remains the gold standard for ATAAD. A variety of risk factors associated with in-hospital and long-term death in patients with aortic dissection have been elucidated (Bonser et al., 2011; Kruger et al., 2011; Benedetto et al., 2021).

Bilirubin is an end-product of heme metabolism and has been increasingly recognized as a potent endogenous antioxidant (Zucker et al., 2001; Zelenka et al., 2016). However, several *in vitro* and *in vivo* studies demonstrated that oxidative stress induced by elevated concentrations of bilirubin can cause bilirubin-induced cell toxicity (Seubert et al., 2002; Yueh et al., 2014; Rawat et al., 2018). A previous study indicated that hyperbilirubinemic Ugt1^−/−^ mice had an incomplete antioxidant defense with activation of key oxidative stress markers (Bortolussi et al., 2015). Another study suggested that hyperbilirubinemia could affect reactive oxygen species homeostasis and participate in the regulation of inflammation, senescence, as well as mitochondrial dysfunction in aged rats (Zelenka et al., 2016).

Hyperbilirubinemia is a common complication that occurs after cardiac surgery due to hemolysis and/or liver hypoperfusion, especially under cardiopulmonary bypass (CPB) (Collins et al., 1983; Wang et al., 1994). Previous studies indicated that the incidence of postoperative hyperbilirubinemia (PH) ranged from 10% to 40% in cardiac surgery and associated with increased postoperative morbidity and mortality (Nishi et al., 2012; Yang et al., 2019; Chen et al., 2021). However, the clinical significance of PH after surgical repair of ATAAD has not been evaluated. In this study, we aimed to identify the incidence, risk factors, and prognosis of PH after ATAAD surgical repair.

## Material and methods

### Study population and demographic characteristics

Consecutive patients diagnosed with ATAAD from January 2014 to December 2019 at Nanjing Drum Tower Hospital were retrospectively enrolled for this study. Patients who received renal replacement therapy at the time of admission (*n* = 27) and/or a total of preoperative bilirubin level greater than 3 mg/dl (*n* = 30) were excluded. In addition, patients who died within 24 h after surgery or with incomplete data were also excluded. Eventually, a total of 970 patients were included in the final analysis. The ethics of Nanjing Drum Tower Hospital approved this retrospective study and waived the requirement of informed consent due to the retrospective nature of this study.

Demographic parameters, comorbidities, and operation details were retrieved from the electronic medical record system. Postoperative PH was defined as total serum bilirubin concentration greater than 3 mg/dl within the first week after the surgery (Collins et al., 1983; Wang et al., 1994; Hsu et al., 2007; Nishi et al., 2012). Patients were divided into the PH group and the control group according to their postoperative bilirubin levels. The acute kidney injury (AKI) was diagnosed based on the Kidney Disease Improving Global Outcomes criteria (Kellum et al., 2012). Central venous pressure (CVP) was measured every 6 h in the first 3 postoperative days *via* the internal jugular vein, and the mean values were recorded.

### Statistical analysis

Categorical variables were presented as frequencies with percentages. Continuous variables were presented as mean ± standard deviation. Chi-square test or Fisher’s exact test was applied to compare categorical variables, whereas Student’s t-test was used for continuous variables. To exclude potential baseline confounders, a 1:1 propensity score matching (PSM) was conducted between two groups. All the pre-operative variables and intro-operative variables were included in the analysis. The standardized differences for variables were balanced post-match. A standardized difference <20% was considered an acceptable imbalance. Logistic regression models were used to find univariable and multivariable risk factors for PH. Variables with *p*-value <0.2 identified in the univariable analysis were further included in the multivariable model determined *via* a stepwise enter procedure. Kaplan-Meier analysis was conducted to assess the association between PH and mortality. Differences of survival rate between groups were further analyzed with log-rank tests. Cox proportional hazard analysis was conducted to explore independent factors that affect long-term survival with variables identified in previous univariable analyses with a *p* value less than 0.20. A forward stepwise procedure was applied to introduce variables to the final models. A *p* value less than 0.05 was considered statistically significant. All analyses were conducted with SPSS version 26 (SPSS Inc., Chicago, IL).

## Results

### Overall cohort

The demographic and clinical characteristics of patients with and without PH were summarized in Table 1. Of the 970 patients included in this study, 717 patients (73.9%) were male. The mean age was 52.9 ± 13.1 years and the mean body mass index was 25.6 ± 4.8 kg/m^2^. The occurrences of comorbidities including hypertension (72.0%), diabetes mellitus (2.4%), coronary artery disease (3.5%), and stroke (3.6%) were summarized and compared between two groups. The PH was diagnosed in 183 of all enrolled patients with an overall incidence of 18.9%. Among patients who were diagnosed with PH, their peak serum total bilirubin concentration was 5.8 ± 3.9 mg/dl. Significant differences of age and occurrence of limb ischemia, cerebral ischemia, coronary ischemia, and pericardial tamponade. As shown in Table 2, significant differences of preoperative triglyceride, total bilirubin, and alanine transferase levels were identified between patients with and without PH.

**TABLE 1:** Comparison of preoperative variables.

Variables	Total (*n* = 970)	Overall cohort			PSM cohort		
		Normal bilirubin (*n* = 787)	Hyperbilirubinemia (*n* = 183)	*p* Value	Normal bilirubin (*n* = 144)	Hyperbilirubinemia (*n* = 144)	*p* Value
DeBakey type I (%)	798 (82.3)	647 (82.2)	151 (82.3)	0.923	121 (84.0)	119 (82.6)	0.752
Demographic data							
Age (year)	52.9 ± 13.1	52.2 ± 13.0	56.3 ± 13.1	<0.001	49.8 ± 12.1	52.0 ± 12.1	0.279
Male (%)	717 (73.9)	589 (74.8)	128 (69.9)	0.174	105 (72.9)	100 (69.4)	0.515
BMI (kg/m2)	25.6 ± 4.8	25.6 ± 4.9	25.8 ± 4.5	0.591	25.0 ± 7.2	25.8 ± 4.7	0.466
Medical history							
Hypertension (%)	698 (72.0)	561 (71.3)	137 (74.9)	0.331	98 (68.1)	108 (75.0)	0.192
Diabetes mellitus (%)	23 (2.4)	21 (2.7)	2 (1.1)	0.284	4 (2.8)	1 (0.7)	0.371
Coronary artery disease (%)	34 (3.5)	26 (3.3)	8 (4.4)	0.479	4 (2.8)	7 (4.9)	0.356
Stroke (%)	35 (3.6)	27 (3.4)	8 (4.4)	0.539	3 (2.1)	6 (4.2)	0.501
Marfan syndrome (%)	27 (2.8)	18 (2.3)	9 (4.9)	0.051	3 (2.1)	8 (5.6)	0.124
Previous cardiac surgery (%)							
PCI (%)	8 (0.8)	5 (0.6)	3 (1.6)	0.178	0 (0)	3 (2.1)	0.247
TEVAR (%)	19 (2.0)	13 (1.7)	6 (3.3)	0.148	5 (3.5)	6 (4.2)	0.759
CABG (%)	1 (0.1)	1 (0.1)	0 (0)	1.000	-	-	-
AVR (%)	15 (1.5)	11 (1.4)	4 (2.2)	0.502	4 (2.8)	4 (2.8)	1.000
Limb ischemia (%)	121 (12.5)	83 (10.5)	38 (20.8)	<0.001	18 (12.5)	21 (14.6)	0.605
Mesenteric ischemia (%)	39 (4.0)	28 (3.6)	11 (6.0)	0.128	6 (4.2)	11 7.6)	0.211
Cerebral ischemia (%)	91 (9.4)	63 (8.0)	28 (15.3)	0.002	11 (7.6)	13 (9.0)	0.670
Coronary ischemia (%)	54 (5.6)	36 (4.6)	18 (9.8)	0.005	8 (5.6)	16 (11.1)	0.088
Pericardial tamponade (%)	157 (16.2)	117 (14.9)	40 (21.9)	0.021	8 (11.0)	15 (20.5)	0.112
LVEF (%)	55.1 ± 6.6	55.1 ± 6.6	54.9 ± 6.7	0.860	57.7 ± 3.1	54.0 ± 7.6	0.276
Involving the celiac trunk (%)	188 (19.4)	145 (18.4)	43 (23.5)	0.118	24 (16.7)	28 (19.4)	0.540
Involving the superior mesenteric artery (%)	231 (23.8)	179 (22.7)	52 (28.4)	0.105	38 (26.4)	44 (30.6)	0.433

Continuous variables are presented as mean ± standard deviation, for categorical variables number (percentage) are shown.

BMI, body mass index; PCI, percutaneous coronary intervention; TEVAR, thoracic endovascular repair; CABG, coronary artery bypass grafting; AVR, aortic valve replacement; LVEF, left ventricular ejection fraction; PSM, propensity score matching.

**TABLE 2:** Comparison of laboratory tests upon admission.

Variables	Total (*n* = 970)	Overall cohort			PSM cohort		
		Normal bilirubin (*n* = 787)	Hyperbilirubinemia (*n* = 183)	*p* Value	Normal bilirubin (*n* = 144)	Hyperbilirubinemia (*n* = 144)	*p* Value
WBC (10^9^/L)	11.7 ± 11.2	11.8 ± 12.3	11.3 ± 4.0	0.585	11.8 ± 4.3	11.3 ± 4.8	0.575
Haemoglobin (g/L)	122.8 ± 29.0	123.2 ± 29.7	121.0 ± 25.7	0.351	123.4 ± 25.1	122.0 ± 25.9	0.734
PLT (10^9^/L)	151.0 ± 86.3	150.6 ± 63.6	152.7 ± 148.6	0.854	152.8 ± 58.0	136.4 ± 64.5	0.110
Fibrinogen (g/L)	2.6 ± 1.4	2.6 ± 1.4	2.5 ± 1.4	0.689	2.5 ± 1.2	2.3 ± 1.2	0.212
Triglyceride (mmol/L)	1.3 ± 1.4	1.4 ± 1.5	1.1 ± 0.8	0.041	1.1 ± 0.6	1.1 ± 0.9	0.961
CRP (mg/dl)	48.3 ± 55.4	47.6 ± 55.9	51.1 ± 53.2	0.489	49.5 ± 59.8	34.0 ± 45.2	0.116
D-dimer (ng/ml)	9.9 ± 17.6	9.2 ± 15.6	12.5 ± 23.8	0.128	12.8 ± 19.8	9.2 ± 10.2	0.267
Albumin (g/L)	36.6 ± 5.0	36.7 ± 4.9	36.1 ± 5.7	0.240	37.2 ± 4.9	38.5 ± 5.1	0.145
TnT (ng/ml)	0.3 ± 0.8	0.3 ± 0.8	0.3 ± 0.7	1.000	0.3 ± 0.6	0.2 ± 0.4	0.168
ALT (U/L)	81.5 ± 241.4	66.4 ± 198.5	147.1 ± 368.2	0.007	46.5 ± 77.5	49.4 ± 78.6	0.834
Bun (mmol/L)	8.2 ± 3.9	8.1 ± 4.0	8.2 ± 3.4	0.791	8.3 ± 3.5	7.6 ± 3.1	0.255
sCr (μmol/L)	108.3 ± 109.7	109.4 ± 117.5	103.7 ± 66.2	0.527	105.7 ± 76.6	93.7 ± 52.1	0.271
Total bilirubin (mg/dl)	2.1 ± 1.3	1.9 ± 1.1	2.8 ± 1.7	<0.001	2.0 ± 0.8	1.8 ± 0.9	0.091
INR	1.2 ± 0.7	1.2 ± 0.7	1.2 ± 0.3	0.350	1.1 ± 0.2	1.2 ± 0.3	0.127

Continuous variables are presented as mean ± standard deviation, for categorical variables number (percentage) are shown.

WBC, white blood cell; PLT, platelet; CRP, c-reactive protein; TnT, troponin T; ALT, alanine transaminase; Bun, blood urea nitrogen; sCr, serum creatinine; INR, international normalized ratio; PSM, propensity score matching.

**FIGURE 1:**
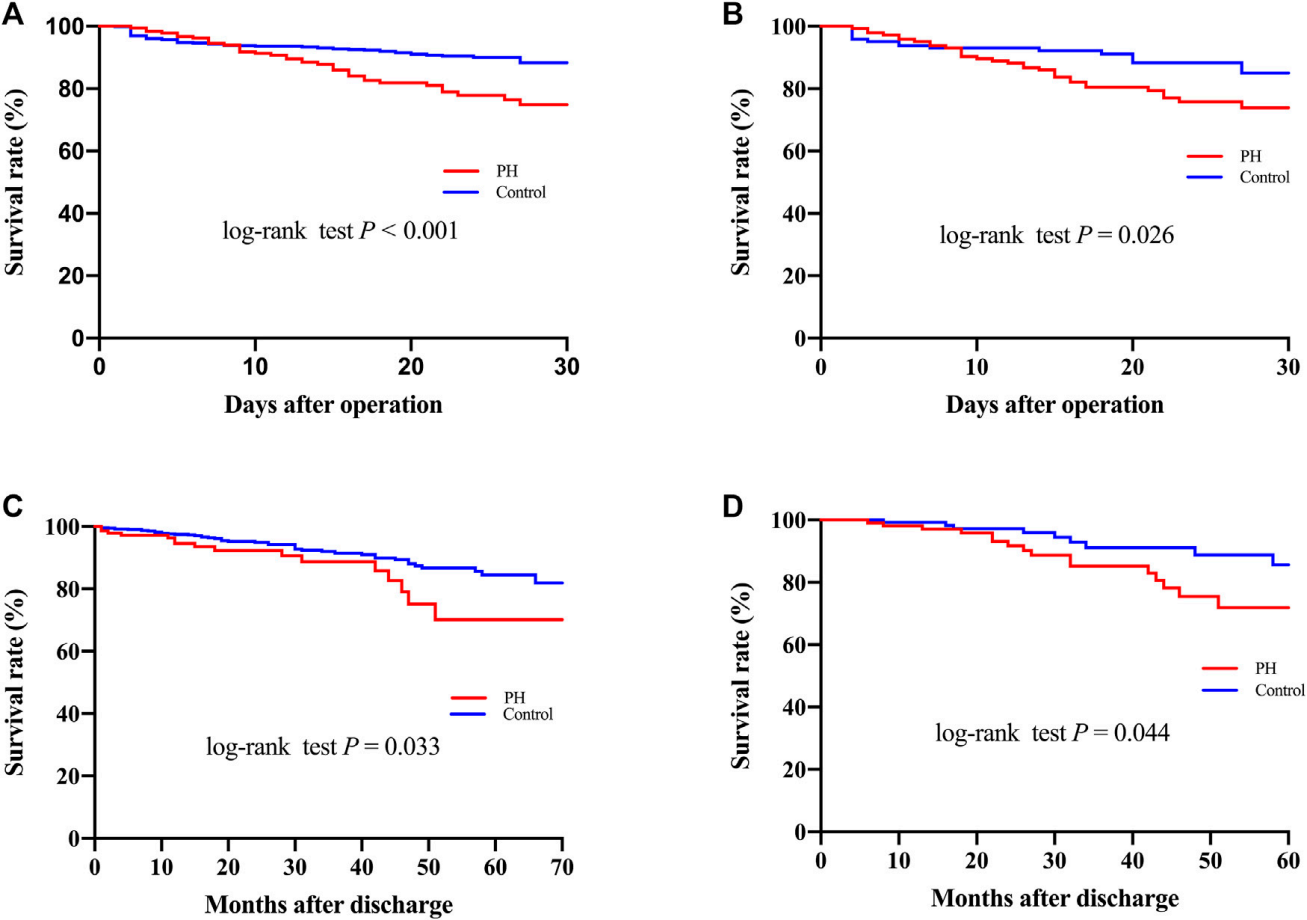
Kaplan–Meier curves **(A–C)** for 30-Day and long-term mortality before **(A,C)** and after **(B,D)** propensity score matching.

Surgical procedure parameters were presented in Table 3 and the data showed significant differences of CPB duration as well as aortic cross-clamp time between two groups. In-hospital outcomes were shown in Table 4. Patients with PH were associated with elevated CVP levels (15.0 ± 5.3 vs. 10.6 ± 4.7, *p* < 0.001) and higher 30-Day mortality (20.8% vs. 9.0%, *p* < 0.001), more frequent postoperative AKI (61.7% vs. 46.5%, *p* < 0.001), hemodialysis (23.5% vs. 13.9%, *p* = 0.001), as well as prolonged intubation duration compared to controls. A significant difference of 30-Day mortality was observed between two groups by the Kaplan-Meier survival curves (Figure 1A; *p* < 0.001 by log-rank test).

**TABLE 3:** Comparison of operative variables.

Variables	Total (*n* = 970)	Overall cohort			PSM cohort		
		Normal bilirubin (*n* = 787)	Hyperbilirubinemia (*n* = 183)	*p* Value	Normal bilirubin (*n* = 144)	Hyperbilirubinemia (*n* = 144)	*p* Value
Concomitant CABG (%)	57 (5.9)	42 (5.3)	15 (8.2)	0.138	5 (3.5)	12 (8.3)	0.080
CPB time (min)	233.8 ± 68.2	230.2 ± 66.3	249.7 ± 73.9	0.001	224.1 ± 56.3	243.1 ± 65.3	0.063
Aortic cross-clamp time (min)	164.3 ± 56.0	161.6 ± 54.1	176.2 ± 62.4	0.004	159.9 ± 49.2	172.0 ± 59.5	0.182
DHCA time (min)	29.4 ± 12.9	29.0 ± 12.8	31.0 ± 13.3	0.068	30.2 ± 12.8	31.7 ± 12.8	0.444
Root procedure							
Bentall (%)	240 (24.7)	193 (24.5)	47 (25.7)	0.743	43 (29.9)	45 (31.3)	0.798
Root reconstruction (%)	695 (71.6)	566 (71.9)	129 (70.5)	0.700	91 (63.2)	94 (65.3)	0.712
Valve sparing root replacement (%)	37 (3.8)	21 (2.7)	16 (8.7)	<0.001	4 (2.8)	7 (4.9)	0.356
Distal surgical technique							
Hemi-arch replacement (%)	207 (21.3)	162 (20.6)	45 (24.6)	0.234	33 (22.9)	38 (26.4)	0.494
Total arch + frozen elephant trunk (%)	460 (47.4)	371 (47.1)	89 (48.6)	0.716	73 (50.7)	68 (47.2)	0.556
Arch fenestrated stent graft (%)	302 (31.1)	245 (31.1)	57 (31.1)	0.997	44 (30.6)	47 (32.6)	0.704

Continuous variables are presented as mean ± standard deviation, for categorical variables number (percentage) are shown.

CABG, coronary artery bypass graft; CPB, cardiopulmonary bypass; DHCA, deep hypothermic circulatory arrest; PSM, propensity score matching.

**TABLE 4:** Comparison of postoperative variables.

Variables	Total (n = 970)	Overall cohort			PSM cohort		
		Normal bilirubin (*n* = 787)	Hyperbilirubinemia (*n* = 183)	*p* Value	Normal bilirubin (*n* = 144)	Hyperbilirubinemia (*n* = 144)	*p* Value
Total bilirubin (mg/dl)	2.3 ± 2.5	1.5 ± 0.6	5.8 ± 3.9	<0.001	1.7 ± 0.6	5.7 ± 3.9	<0.001
unconjugated bilirubin (mg/dl)	0.9 ± 0.9	0.6 ± 0.4	2.2 ± 1.5	<0.001	0.7 ± 0.3	2.1 ± 1.5	<0.001
conjugated bilirubin (mg/dl)	1.4 ± 1.6	0.9 ± 0.4	3.6 ± 2.5	<0.001	1.0 ± 0.3	3.6 ± 2.5	<0.001
Postoperative complications (%)							
Re-exploration for bleeding (%)	39 (4.0)	28 (3.6)	11 (6.0)	0.128	10 (6.9)	10 (6.9)	1.000
Dialysis (%)	152 (15.7)	109 (13.9)	43 (23.5)	0.001	26 (18.1)	35 (24.3)	0.194
AKI (%)	479 (49.4)	366 (46.5)	113 (61.7)	<0.001	66 (45.8)	97 (67.4)	<0.001
Stroke (%)	78 (8.0)	62 (7.9)	16 (8.7)	0.698	10 (6.9)	13 (9.0)	0.514
Paraplegia (%)	31 (3.2)	25 (3.2)	6 (3.3)	0.944	4 (2.8)	4 (2.8)	1.000
Tracheostomy (%)	43 (4.4)	32 (4.1)	11 (6.0)	0.250	9 (6.3)	8 (5.6)	0.803
Deep sternal wound infection (%)	13 (1.3)	9 (1.1)	4 (2.2)	0.282	1 (0.7)	4 (2.8)	0.371
CVP (cmH_2_O)	11.4 ± 5.1	10.6 ± 4.7	15.0 ± 5.3	<0.001	10.7 ± 4.4	11.3 ± 4.5	0.423
Drainage volume 24 h after surgery (ml)	720.1 ± 649.2	702.0 ± 649.7	798.7 ± 642.7	0.081	703.6 ± 813.0	820.9 ± 669.6	0.349
Ventilation time (hour)	38.6 ± 57.8	35.1 ± 49.7	57.7 ± 88.1	0.011	29.3 ± 39.2	62.0 ± 95.6	0.027
ICU stay (day)	6.7 ± 10.0	6.4 ± 10.5	7.9 ± 6.9	0.068	5.7 ± 4.6	7.6 ± 6.0	0.036
Hospital stay (day)	21.3 ± 12.2	20.9 ± 11.7	23.3 ± 14.3	0.031	19.6 ± 9.1	25.4 ± 13.9	0.003
30-Day mortality (%)	109 (11.2)	71 (9.0)	38 (20.8)	<0.001	15 (10.4)	32 (22.2)	0.007

Continuous variables are presented as mean ± standard deviation, for categorical variables number (percentage) are shown.

AKI, acute kidney injury; CVP, central venous pressure; ICU, intensive care unit; PSM, propensity score matching.

**FIGURE 2:**
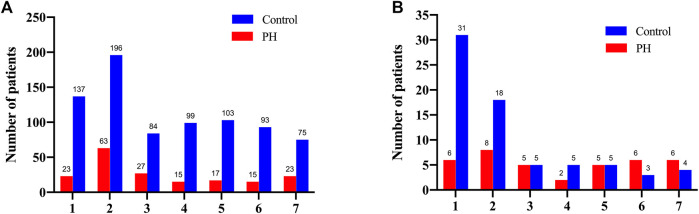
The number of the overall patients **(A)** and the deceased patients **(B)** according to the postoperative days of peak bilirubin level for the both groups.

Our data indicated that the serum bilirubin level was peaked in 333 patients (42.3%) within the first 2 days after surgery in the control group. In the PH group, the level peaked on the 2nd postoperative day in 63 patients (34.4%) and on the 1st postoperative day in 23 (12.6%) patients (Figure 2A). Interestingly, we noticed that the patients in the control group whose serum bilirubin level peaked on the 1st and 2nd postoperative day were associated with significant increase of 30-Day mortality. Similarly, among all 38 patients in the PH group who died within 30 days, 8 had peak bilirubin levels on the 2nd postoperative day and 6 on the 1st postoperative day (Figure 2B).

Our results also showed 18.6% (*n* = 34) of patients with PH had elevated unconjugated-dominant bilirubin. Conjugated- and unconjugated-bilirubin levels showed different changing patterns, with most of cases (81.4%) shown to be conjugated-bilirubin-dominant. The 30-Day mortality rate was significantly higher in patients with conjugated-bilirubin-dominant PH than in patients with unconjugated bilirubin (25.5% vs. 0%, *p* < 0.001).

**FIGURE 3:**
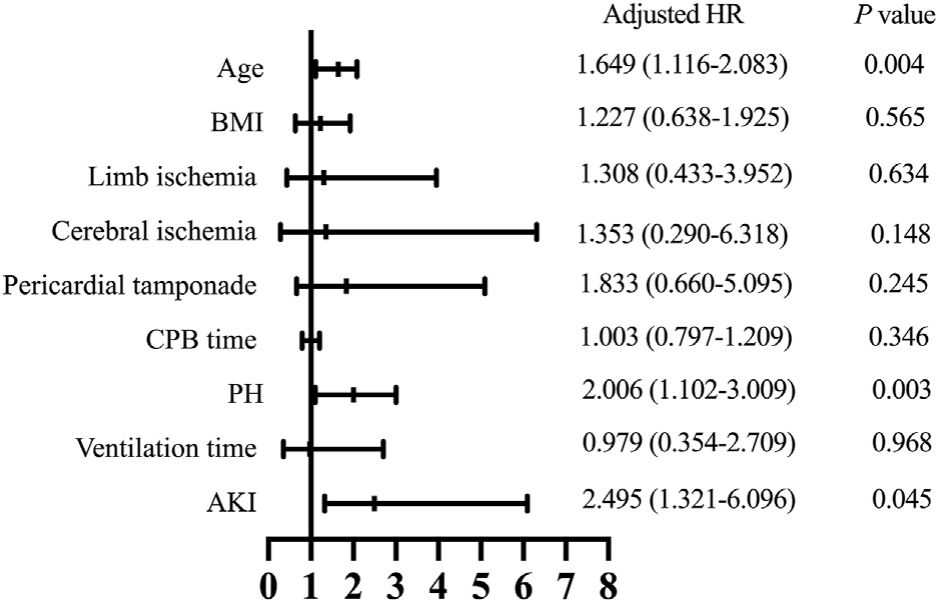
Hazard ratios for long-term survival. BMI, body mass index; CPB, cardiopulmonary bypass; PH, post hyperbilirubinemia; AKI, acute kidney injury; HR, hazard ratio.

Follow-up was conducted by telephone call once every year, and it was completed in 919 patients (94.7%) with a median follow-up time of 36 months. The patients who were lost to follow-up were identified as censored data in the outcome analysis. A total of 52 patients in the PH group and 113 in the control group died during the follow-up. As shown in Figure 1C, the Kaplan-Meier analysis indicated that the mortality was significantly correlated with the development of PH (log-rank test, *p* = 0.033). After adjusting for confounders, the hazard ratio for PH was 2.006 (95% confidence interval [CI]: 1.102–3.009, *p* = 0.003) and was significantly associated with worse long-term survival (Figure 3).

### Propensity score-matched cohort

After PSM, 144 pairs of patients with similar baseline characteristics and operative variables were identified. The 30-Day mortality rates were 22.2% in the PH group and 10.4% in the control group (*p* = 0.007) (Table 4). Postoperative AKI was more frequently observed in patients with PH (67.4% vs. 45.8%, *p* < 0.001). In addition, the ventilation duration, average intensive care unit stay, and hospital stay were significantly prolonged in the PH group. Similarly, Figure 1B showed that the Kaplan-Meier survival curves identified a significant association between PH and 30-Day mortality (log-rank test, *p* = 0.026). In addition, the long-term survival rate was also significantly decreased in patients with PH, compared to patients in the control group (log-rank test, *p* = 0.044) (Figure 1D).

### Risk factors for PH

**FIGURE 4:**
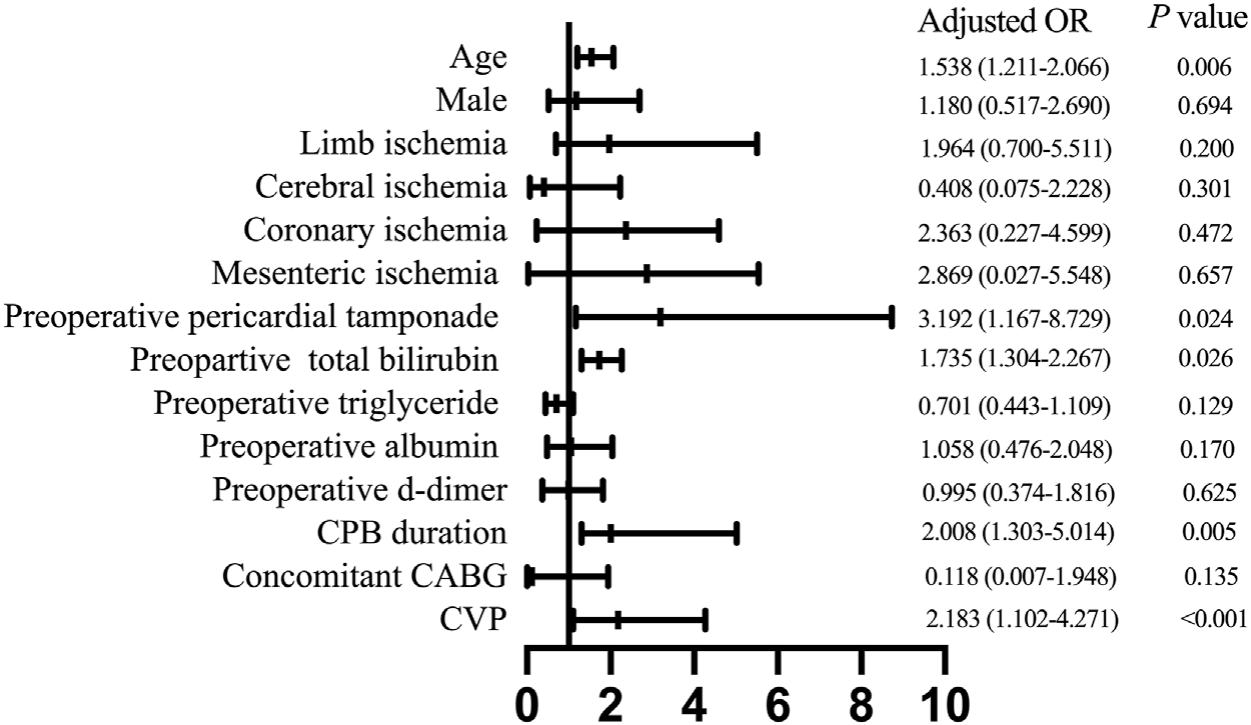
Multivariate analysis of risk factors for postoperative hyperbilirubinemia. CPB, cardiopulmonary bypass; CABG, coronary artery bypass grafting; CVP, central venous pressure.

Next, we conducted a multivariable analysis and found out that advanced age [odds ratio (OR) 1.538, 95% confidence interval (CI) 1.211–2.066; *p* = 0.006], preoperative pericardial tamponade (OR 3.192, 95% CI 1.167–8.792; *p* = 0.024), increased preoperative total bilirubin (OR 1.735, 95% CI 1.304–2.267; *p* = 0.026), prolonged CPB duration (OR 2.008, 95% CI 1.303–5.014; *p* = 0.005), and elevated postoperative CVP level (OR 2.183, 95% CI 1.102–4.271; *p* < 0.001) were independent risk factors for developing PH after ATAAD surgery (Figure 4).

## Discussion

Even though the development of PH is relatively common after ATAAD surgery, how it affects the prognosis remain elusive. The present study not only identified the known association between PH development and prognosis, but also the independent risk factors for PH development after receiving ATAAD surgical repair. The results demonstrated that the occurrence of hyperbilirubinemia after ATAAD surgical repair was common and associated with increased hospital stay and intensive care unit stay. The short- and long-term mortality was significantly increased in patients with PH compared to those without PH.

Our data showed that 18.9% of all patients enrolled in this study developed PH and the occurrence was similar to the data reported in a previous study conducted in patients who received modern extracorporeal circulation treatment (An et al., 2006). Hemolysis induced hyperbilirubinemia can occur after CPB due to the use of cardiotomy suction, membrane oxygenator, and various other procedures (Garcia et al., 1996). In addition, the non-pulsatile blood supply in CPB may cause regional malperfusion and result in liver cell damage (Hornick and Taylor, 1997). Furthermore, hemodilution induced by CPB can further reduce tissue oxygen delivery and contribute to ischemic damage.

It has been reported that severe hyperbilirubinemia was associated with increased morbidity and mortality in patients underwent cardiac surgery (Michalopoulos et al., 1997). Our findings demonstrated that the development of PH was associated with a more than two-fold increase of 30-Day mortality after receiving ATAAD surgery. This result was consistent with a previous report showing that mortality among patients with PH was as high as 19.0% (Nishi et al., 2012). The current study showed that the occurrence of postoperative AKI increased from 49.4% in all patients to 67.4% in those with PH. It is important to point out that similar observation was also discovered in a previous study conducted in patients who received cardiac surgeries (Kraev et al., 2008). Mechanism study showed that hyperbilirubinemia has pro-apoptotic effects and can aggravate renal ischemia-reperfusion injury (Yuan et al., 2019). In addition, high concentration of bilirubin could induce inflammatory response and cell apoptosis in brain (Barateiro et al., 2014), which might be another potential mechanism contributes to the high mortality identified in our present cohort.

Similar to previous studies, we found that the PH was a strong independent predictor for increasing long-term mortality in patients who received ATAAD surgery (Kraev et al., 2008; Kruger et al., 2011). The predictive nature of hyperbilirubinemia on long-term outcomes was identified in multivariable logistic regression analysis, after adjusting for major pre-, intra-, or postoperative comorbidities. It is important to point out that this study was not powered to differentiate the causes of hyperbilirubinemia which might provide further information regarding its predictive value.

Our study suggested that majority of patients had peak bilirubin levels within first 2 days after surgery in the control group, which was consistent with a report from Collins and associates (Collins et al., 1983). The development of liver failure has long been known to be associated with increased morbidity and mortality. We proposed that the persistent increasing levels of bilirubin following cardiac surgery may occur before liver injury. On the other hand, the spontaneous occurrence of PH and rapid decline may associate with the transient liver damage occurred during CPB. Currently, limited treatments have been explored to treat the PH after ATAAD surgery. Bilirubin adsorption treatment have shown promising results after cardiac surgery in our center (Pan et al., 2021) and might provide an option.

The postoperative hyperbilirubinemia can be induced by various reasons. Another merit of this study was the identification of independent risk factors for the development of PH. Preoperative pericardial tamponade was found to be one of the risk factors. Pericardial tamponade could lead to hypotension in ATTAD patients. The blood supply of visceral organ is maintained by proper arterial blood pressure. Due to its dual blood supply, liver is relatively resistant to necrosis under hypoperfusion and hypoxia conditions. However, hepatic necrosis may occur upon severe or prolonged hypoperfusion and result in impaired liver function (Hessel, 2004). Sabzi and Faraji (2015) reported in their study that the occurrence of hypotension was associated with alkaline phosphatase and aspartate aminotransferase changes in patients who received cardiac surgery. Aortic dissections may involve coeliac trunk artery and result in liver hypoperfusion that can be further deteriorated by preoperative induced hypotension. However, increasing blood pressure before the surgery may increase the risk of aortic rupture or lesion progression. Therefore, appropriate arterial blood pressure control before surgery is critical.

Duration of CPB was identified as a predictor of elevated bilirubin which was constant with previous studies (Wang et al., 1994; Kraev et al., 2008). Several reports have shown that CPB can induce liver damage (Kraev et al., 2008; Yang et al., 2019) and our previous study proved that the prolonged CPB duration was a risk factor for postoperative hepatic dysfunction in aortic dissection repairs (Wang et al., 2021). Among all independent risk factors for PH, CPB duration and CVP were the only 2 factors that could be modified. For instance, advances in surgical technique, proper regulation and control of CPB temperature, and a skilled surgical team could shorten the CPB duration, reduce CPB-related inflammation and decrease hepatic ischemia-reperfusion injury.

Postoperative elevated CVP level might be associated with a “congested” state of the liver, which might lead to inappropriate oxygen delivery and energy deficit that eventually lead to the impairment of its capacity to dispose bilirubin (Chu et al., 1984). One previous study found that preoperative congestive heart failure was an independent risk factor for PH, which was consistent with our discoveries (Kraev et al., 2008). Elevated postoperative CVP level might be attributed to the right ventricular dysfunction. It is important to point out that this was the first study identified early postoperative CVP as a predictor for the development of PH after extracorporeal circulation surgery.

Both unconjugated and conjugated hyperbilirubinemia can occur in postoperative PH. Early postoperative unconjugated hyperbilirubinemia may be results of CPB induced intravascular haemolysis, mechanical valve leak or extravascular haemolysis of transfused red blood cells which often not used to estimate liver injury. Conjugated hyperbilirubinemia may result from cholestasis caused by hepatic ischemic injury and predisposes patients to cholangitis (Diaz and Renz, 2014; Yang et al., 2019). Our data showed that both unconjugated bilirubin and conjugated bilirubin increased substantially in patients after receiving surgical repair, regardless of the presence of PH. The increase of conjugated bilirubin was more prominent than unconjugated bilirubin in our study, which further indicated the involvement of hepatic injury.

This study has several limitations. Firstly, this was a retrospective study conducted in a single center that might not be representable for the general population. In addition, no consensus of PH definition has been reached when this study was being conducted therefore it might be difficult to compare the result of this study to others. Furthermore, some parameters such as preoperative CVP, the amount of blood transfusion volume, the presence of viral hepatitis or drug-induced hepatitis and anesthetic-related liver dysfunction were not measured in the study and may limit our findings (Batin et al., 1995; Hirata et al., 1999; Lau et al., 2002). Finally, the surgical technique has evolved over the study period which might also affect the result.

## Conclusion

Our study indicated that PH was commonly observed in patients undergoing ATAAD surgery and associated with increased mortality rate. Age, preoperative total bilirubin level, preoperative pericardial tamponade, CPB duration as well as postoperative CVP level were identified as independent predictors for the development of PH and should be carefully addressed.

## Data Availability

The raw data supporting the conclusions of this article will be made available by the authors, without undue reservation.

## Abbreviations

PH, postoperative hyperbilirubinemia; ATAAD, acute type A aortic dissection; CPB, cardiopulmonary bypass; CVP, central venous pressure; AKI, acute kidney injury; OR, odds ratio; CI, confidence interval.
